# Molecular Tools for qPCR Identification and STR-Based Individual Identification of *Panthera pardus* (Linnaeus, 1758)

**DOI:** 10.3390/genes17010045

**Published:** 2025-12-31

**Authors:** Karolina Mahlerová, Lenka Vaňková, Daniel Vaněk

**Affiliations:** 1 Institute for Environmental Studies, Charles University, 128 01 Prague, Czech Republic; 2 Forensic DNA Service, 170 00 Prague, Czech Republic; 3 Department of Ecology, Faculty of Environmental Sciences, Czech University of Life Sciences, 165 00 Prague, Czech Republic; 4 Department of Legal Forensic Medicine, Bulovka University Hospital, 180 00 Prague, Czech Republic; 5 Department of Forensic Medicine, Second Faculty of Medicine, Charles University, 120 00 Prague, Czech Republic

**Keywords:** *Cytochrome b*, CITES, wildlife trade

## Abstract

**Background/Objectives** The leopard (*Panthera pardus*), an apex predator listed in CITES Appendix I and classified as Vulnerable by the IUCN, is undergoing severe population declines driven by habitat loss, human–wildlife conflict, and illegal trade. Rapid and reliable species and individual identification is critical for conservation and forensic applications, particularly when analyzing highly processed or degraded seized wildlife products, where morphological identification is often impossible. We aimed to develop and validate a robust multiplex quantitative real-time PCR (qPCR) assay combined with a short tandem repeat (STR) system for the species-specific detection and individual identification of *P. pardus*. **Methods** The qPCR assay (Ppar Qplex) was designed to target a mitochondrial Cytochrome b (Cyt b) fragment for species confirmation, a nuclear marker (PLP) for general Feliformia detection and quantification, and an artificial internal positive control (IPC) to monitor PCR inhibition. The assay’s performance was validated for robustness, specificity, sensitivity, repeatability, and reproducibility, utilizing DNA extracted from 30 *P. pardus* individuals (hair and feces) and tested against 18 related Feliformia species and two outgroups. Individual identification was achieved using a set of 18 STR loci and a sex determination system adapted from previously published Panthera panels. **Results** Validation demonstrated high specificity for the Ppar Qplex: mitochondrial amplification occurred exclusively in *P. pardus* samples. The nuclear marker consistently amplified across all 18 tested Feliformia species but not the outgroups. The assay showed high analytical sensitivity, successfully detecting DNA at concentrations as low as 1 pg/µL, with consistent results confirmed across different sample types, replicates, and independent users. Furthermore, the STR multiplex successfully generated 30 unique individual profiles using the 18 polymorphic loci and the sex determination system. **Conclusions** The combined qPCR assay and STR system provide a fast, sensitive, and highly specific molecular framework for rapid leopard detection, quantification, and individual identification from a wide range of sample types. These tools strengthen forensic capacity to combat wildlife crime and provide critical data to support evidence-based conservation management of *P. pardus*. *P. pardus*, an apex predator listed in CITES Appendix I and classified as Vulnerable by the IUCN, is undergoing severe population declines driven by habitat loss, human–wildlife conflict, and illegal trade. Rapid and reliable identification of seized specimens is therefore critical for conservation and forensic applications, mainly when products are highly processed. We developed and validated a multiplex quantitative real-time PCR (qPCR) assay targeting the mitochondrial gene *Cytochrome b* (Cyt b) for species-specific detection. The assay was tested on verified leopard individuals and validated across 18 Feliformia and two outgroup species (*Homo sapiens*, *Canis lupus familiaris*). Analytical performance was assessed through robustness, specificity, sensitivity, repeatability, and reproducibility. Mitochondrial amplification occurred exclusively in leopard samples, while nuclear markers amplified consistently across Feliformia but not in outgroup species. The assay’s limit of DNA detection is 1 pg/µL and produces consistent results across replicates, tested types of samples (hair, feces), and independent users, with internal controls confirming the absence of inhibition. In addition, we present the results of successful individual identification using the set of 18 STR loci and the sex determination system. The developed qPCR and STR systems provide a fast, sensitive, and specific solution for leopard detection and quantification, reinforcing forensic efforts against wildlife crime and supporting conservation of *P. pardus*.

## 1. Introduction

The leopard (*P. pardus*), an apex predator with a wide distribution across Africa and Asia, is listed in Appendix I of the Convention on International Trade in Endangered Species of Wild Fauna and Flora (CITES) and classified as Vulnerable by the International Union for Conservation of Nature (IUCN) [1,2,3]. There are eight recognized subspecies of *P. pardus*—*Panthera pardus pardus* (Sub-Saharan Africa), *P. p. fusca* (Indian subcontinent), *P. p. melas* (Java, Indonesia), *P. p. nimr* (Arabian Peninsula), *P. p. saxicolor* (syn. *tulliana;* Turkey, Iran, Caucasus region), *P. p. orientalis* (Russian Far East, Northeastern China), *P. p. kotiya* (Sri Lanka), and *P. p. delacouri* (Mainland Southeast Asia) [4].

Despite its broad range of geographical distribution and ecological versatility, inhabiting environments from grassland plains and deserts to alpine areas and even the fringes of large cities [1,5,6,7,8], the species is undergoing substantial population declines, consequently leading to a patchier distribution of the previously continuous area of distribution [1,7]. The decline is driven by a combination of factors, including habitat loss and fragmentation, human–wildlife conflict, prey depletion, and the pressures associated with expanding human populations [9,10,11,12]. Furthermore, like other *Panthera* species (*Panthera tigris*, *P. leo*, *P. onca*, *P. uncia*) also listed in Appendix I [2], leopards are impacted by overharvesting and illegal wildlife trade [11]. Their body parts are exploited for ceremonial use, traditional Chinese medicine (TCM) [2,13,14,15,16,17], and poorly managed trophy hunting [18]. These threats remain active and pervasive, with little evidence of mitigation, suggesting a continued risk of population decline without targeted conservation interventions [1,6]. Illegal trade represents a significant but poorly quantified threat to leopard populations. It is estimated that 4500–7000 individuals are harvested annually for their skins, which are widely used in traditional and cultural regalia, particularly in parts of Africa [19]. Approximately 5000 skins, carcasses, stuffed, and live leopards were seized across Asia from 2000 to 2018 [20]. Leopard bones increasingly substitute for tiger bones in traditional Asian medicine, and claws and teeth are traded as amulets or curios, highlighting the species’ exploitation across multiple markets [21,22].

Moreover, leopards represent the most frequently reported carnivore involved in human–wildlife conflict globally; their broad ecological niche and adaptability, including a flexible diet and tolerance of human presence, make them particularly prone to conflict with people, as they readily prey on livestock and occasionally threaten human safety [23,24,25,26,27]. This adaptability, which enables leopards to utilize agricultural lands and even urban environments more than any other large carnivore [1,7], further increases exposure to anthropogenic threats, including conflict-driven persecution and illegal hunting for trade. Therefore, rapid and reliable identification of leopards is essential for both conservation research and forensic investigations aimed at combating wildlife crime.

Traditional species and individual identification approaches have relied on morphological traits, such as coat pattern recognition from camera-trap images, pugmark analysis, or morphometric assessment of skulls, claws, hair, and teeth [28,29,30,31,32,33,34,35,36]. While these methods have proven valuable for ecological and conservation monitoring, including population estimation and movement tracking [35,37], they are limited when applied to confiscated wildlife products, which often consist of isolated or processed parts such as powders, bone parts, or TCM [21,22,38,39]. Molecular tools offer robust alternatives while also supporting and enhancing traditional approaches [40]. Mitochondrial DNA barcoding (e.g., Cyt b, COI, 16S) has been extensively used to confirm species identity from degraded or processed samples [40,41,42,43]. The DNA barcodes target standardized regions with interspecific variability exceeding intraspecific variability, enabling species discrimination through comparison with reference databases such as GenBank or the Barcode of Life Data System [40,41,42]. Furthermore, nuclear markers such as microsatellites (STRs) and single-nucleotide polymorphisms (SNPs) allow not only species confirmation but also individual identification, population assignment, and geographic origin tracing [21]. Such molecular approaches are now standard in wildlife forensics and conservation genetics, providing critical evidence in law enforcement and a foundation for effective population management.

Real-time quantitative PCR (qPCR) is a potent tool for wildlife forensics, offering rapid, sensitive, and cost-efficient species detection [44,45,46]. Previous leopard assays have relied on end-point PCR without DNA quantification, lacked inhibition controls [45], and often used amplicons too large for degraded forensic samples [47,48,49], limiting their applicability. We developed a multiplex qPCR assay specifically for *P. pardus* that combines mitochondrial DNA amplification for species confirmation, combined with a nuclear DNA marker for broader detection of Feliformia. The assay is enabling reliable detection from low-concentration samples while simultaneously assessing sample quality through the inclusion of an internal positive control (IPC) and quantity for downstream analyses. The assay is complemented by an individual identification STR system based on 14 nuclear loci and a sex determination system, extending its application beyond species confirmation to population-level monitoring and utilizing already existing *Panthera* genus multiplexes. Together, these tools provide a robust framework for rapid, specific, and sensitive leopard identification, enhancing the capacity to investigate illegal trade and supporting evidence-based conservation management of *P. pardus*.

## 2. Materials and Methods

### 2.1. Specimens Used for the Analyses

The development of a quantification system using real-time PCR and STR plex was tested on 30 *P. pardus* individuals (18 females and 12 males) using hair samples. Eight samples came from *P. p. kotiya*, three from *P. p. orientalis*, and two from *P. p. saxicolor.* The remaining analyzed samples were marked as *P. pardus* without any subspecies information. Furthermore, the real-time PCR quantification plex (*Ppar Qplex*) was tested for specificity on an additional 18 species of Feliformia (*Acinonyx jubatus*, *Caracal caracal*, *Cryptoprocta ferox*, *Civettictis civetta*, *Felis catus*, *Herpailurus yagouaroundi*, *Leopardus tigrinus*, *Leopardus weidii*, *Leptailurus serval*, *Lynx lynx*, *Lynx rufus*, *Otocolobus manual*, *Panthera leo*, *Panthera onca*, *Panthera tigris*, *Panthera uncia*, *Prionailurus bengalensis*, and *Puma concolor*) (source material hair and feces). Hair and feces samples were provided by the Czech zoological gardens and the Czech Environmental Inspectorate. The protection of animals used for scientific purposes, as stated by Directive 2010/63/EU of the European Parliament and of the Council of 22 September 2010, was fully respected. No animal was harmed for the purpose of sample collection. Hair samples were stored dry at ambient temperature before DNA extraction, and feces were stored in DNA/RNA Shield (Zymo Research, Irvine, CA, USA). DNA was extracted from fecal and hair samples using the Quick-DNA Microprep Plus Kit (Zymo Research, USA) following the manufacturer’s protocol. The extracted DNA was quantified using Qubit 4 Fluorometer (ThermoFisher Scientific, Waltham, MA, USA) prior to further analysis.

### 2.2. Quantification System and Species Identification (Ppar Qplex)

Species-specific primers targeting the mtDNA fragment of Cyt b of *P. pardus* were manually designed using BioEdit [50] from publicly available sequences of 16 species of Feliformia, including five individuals of *P. pardus* obtained from NCBI [51,52] and tested against NCBI BLASTn, web interface [53]. The TaqMan probe was manually designed using Primer Express v3.0.1 (ThermoFisher Scientific, Waltham, MA, USA) and tested against NCBI BLAST [53] for specificity. The existing *Llyn Qplex* [46] was adapted to create the *P. pardus*-specific qPCR multiplex by replacing the mitochondrial target with a newly designed Cyt b primer–probe set. The *P. pardus* specific primers and TaqMan probe were evaluated alongside the original Feliformia-specific nuclear marker (PLP) and internal positive control (IPC) primers and TaqMan probes using the Multiple Primer Analyzer tool (Thermo Fisher Scientific, Waltham, MA, USA) to detect potential primer–dimer interactions prior to multiplex optimization (Table 1). The mitochondrial Cyt b gene was selected as the assay target due to its high interspecific variability, strong phylogenetic signal within mammals, and demonstrated suitability for forensic species identification from degraded DNA [54,55].

The qPCR reaction was performed in a total volume of 10 μL consisting of 5 μL of 2× TaqMan Multiplex Master Mix (ThermoFisher Scientific, Waltham, MA, USA), 0.5 μL of 20× qPpar mtDNA Assay mix, 0.5 μL of 20× qPpar nDNA Assay mix, 0.5 μL of 20× qPpar IPC [46], 1 μL of IPC 0.1 pg/μL, 1.5 μL of DNase/RNase-Free Water (Zymo Research, Irvine, CA, USA), and 1 μL of DNA template. Amplification was conducted on QuantStudio™ 5 Real-Time PCR System (ThermoFisher Scientific, Waltham, MA, USA) under the following cycling conditions: 95 °C 20 s; 50× 95 °C 10 s; 60 °C 25 s. The data were analyzed using Design & Analysis Software v2.8.0 (ThermoFisher Scientific, Waltham, MA, USA). Each run included a positive control (a selected individual of *P. pardus* used consistently across all test runs), a negative control consisting of DNase/RNase-Free Water (Zymo Research, Irvine, CA, USA), and other Feliformia species to test specificity. All reactions were performed in technical duplicates per sample to ensure reproducibility.

### 2.3. Validation of the Ppar Qplex

The validation of the *Ppar Qplex* assay included assessments of robustness, specificity, sensitivity, repeatability, and reproducibility. Robustness was evaluated by modifying the annealing temperature. Specificity was tested across 20 species. Sensitivity was assessed using a dilution series of *P. pardus* DNA. Repeatability was evaluated using multiple individuals of *P. pardus*, and reproducibility was examined by comparing assay performance between two laboratory technicians.

### 2.4. STRplex Design and Individual Identification

DNA profiles of *P. pardus* individuals were generated using multiplex STR panels and allelic ladders originally designed for *P. tigris* [56] and *P. leo* [57]. The original STR marker nomenclature was retained to maintain consistency and clarity, with “Ptig” denoting loci derived from the *P. tigris* panel and “Pleo” for those from the *P. leo* panel. The PCR amplification conditions and capillary electrophoresis parameters closely followed previously published protocols for both *P. tigris* and *P. leo* [56,57]. Fragment analysis was conducted via capillary electrophoresis using the SeqStudio™ 3200 Genetic Analyzer System (ThermoFisher Scientific, USA). The data were subsequently analyzed using the genotyping software GeneMapper v5 (ThermoFisher Scientific, USA). Each run included a positive control (a selected individual of *P. pardus* used consistently across all test runs) and a negative control consisting of DNase/RNase-Free Water (Zymo Research, USA).

## 3. Results

### 3.1. Quantification and Species Identification

**Figure 1:**
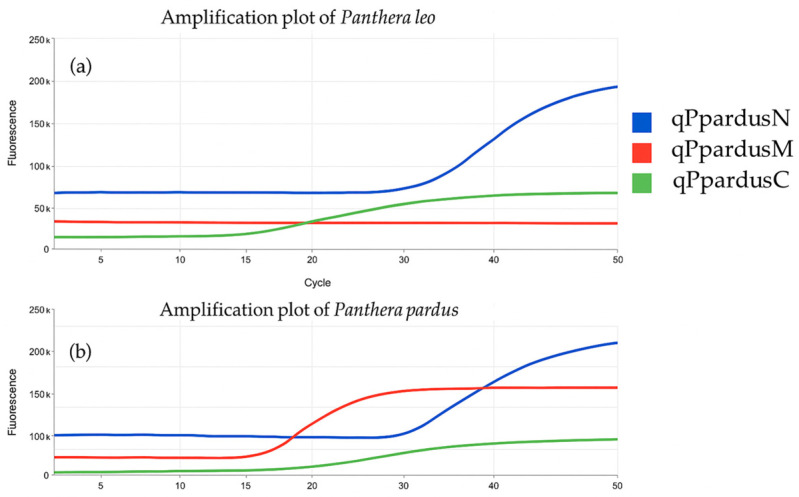
Quantitative real-time PCR (*Ppar Qplex*) amplification curves targeting mitochondrial and nuclear DNA. The assay detects *P. pardus*-specific mitochondrial DNA (qPpardusM; red), Feliformia-specific nuclear DNA (qPpardusN; blue), and an internal positive control (qPpardusC; IPC; green): (**a**) Amplification from *P. leo* DNA extract shows nuclear and IPC signals but no amplification of the *P. pardus*-specific target (qPpardusM Ct: Underdetection, qPpardusN Ct: 33, qPpardusC Ct: 21). (**b**) Amplification from *P. pardus* DNA extract, showing positive signals for all three targets (qPpardusM Ct: 21, qPpardusN Ct: 31, qPpardusC Ct: 20).

*P. pardus* was identified using quantitative real-time PCR using the *Ppar Qplex*. Amplification of a 164 bp fragment of the mitochondrial Cyt b gene (red curve) confirmed the presence of *P. pardus* DNA in the sample. Amplification of a 132 bp Feliformia fragment of the proteolipid protein (PLP) gene (blue curve) provides broader taxonomic confirmation. The internal positive control (IPC), a synthetic 261 bp fragment, was detected via the green curve and confirmed reaction integrity while flagging potential PCR inhibition (Figure 1). Concurrent amplification of all three targets (Figure 1) confirms species identity, taxonomic relevance, and sample quality. Furthermore, DNA was quantified using the *Ppar Qplex* quantification system based on four standards: S1: 0.06 ng/μL nDNA; S2: 0.012 ng/μL nDNA, S3: 0.0024 ng/μL nDNA; and S4: 0.00048 ng/μL nDNA.

### 3.2. Results of Validation of the Ppar Qplex

#### 3.2.1. Robustness

Modifying the annealing temperature tested the assay’s robustness. The optimal temperature was 60 °C. Deviations of ±2 °C did not result in failure, and all targets (Cyt b, PLP, IPC) were amplified. Deviations greater than ±4 °C failed to amplify targets, demonstrating the assay’s thermal sensitivity.

#### 3.2.2. Specificity

All samples were diluted to 1 ng/µL and tested across 20 species. Nuclear DNA amplification was observed exclusively in Feliformia species (18 tested species), with no amplification in outgroup species (*H. sapiens*, *C. l. familiaris*). Mitochondrial DNA was amplified solely in Panthera pardus, confirming the high specificity of the mtDNA primers; nDNA was amplified across all Feliformia species and was not amplified in the two outgroup species.

#### 3.2.3. Sensitivity

DNA extracted from *P. pardus* was diluted to concentrations of 1 pg/µL, 10 pg/µL, 100 pg/µL, and 1 ng/µL. Both mitochondrial and nuclear markers, as well as an internal positive control, were successfully amplified across all dilutions. Analytical sensitivity was further evaluated using serial dilutions (1 pg/µL, 10 pg/µL, and 100 pg/µL) of DNA from eight related species (*A. jubatus*, *C. caracal*, *F. catus*, *L. serval*, *L. lynx*, *P. leo*, *P. tigris*, and *P. concolor*). Amplification of nDNA was successful across all concentrations.

#### 3.2.4. Repeatability

Assay repeatability was assessed using DNA extracts from 4 *P. pardus* individuals tested in triplicate. All replicates, including negative controls, showed consistent results, confirming assay repeatability.

#### 3.2.5. Reproducibility

The assay was independently performed by two laboratory technicians using the same set of 10 samples. Results were consistent across all runs, verifying reproducibility across users and sessions.

### 3.3. Individual Identification

**Figure 2:**
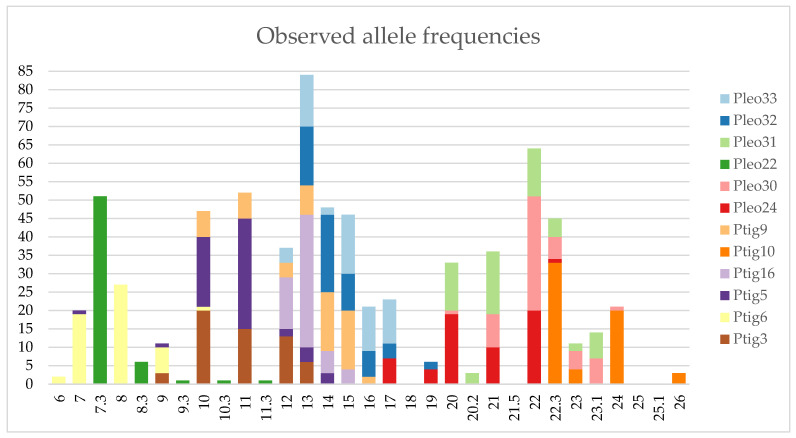
Allele frequencies (Y-axis: total number of observations per allele) observed in 30 *P. pardus* individuals across STR loci Ptig3, Ptig6, Ptig5, Ptig16, Ptig10, Ptig9, Pleo24, Pleo30, Pleo22, Pleo31, Pleo32, and Pleo33.

**Figure 3:**
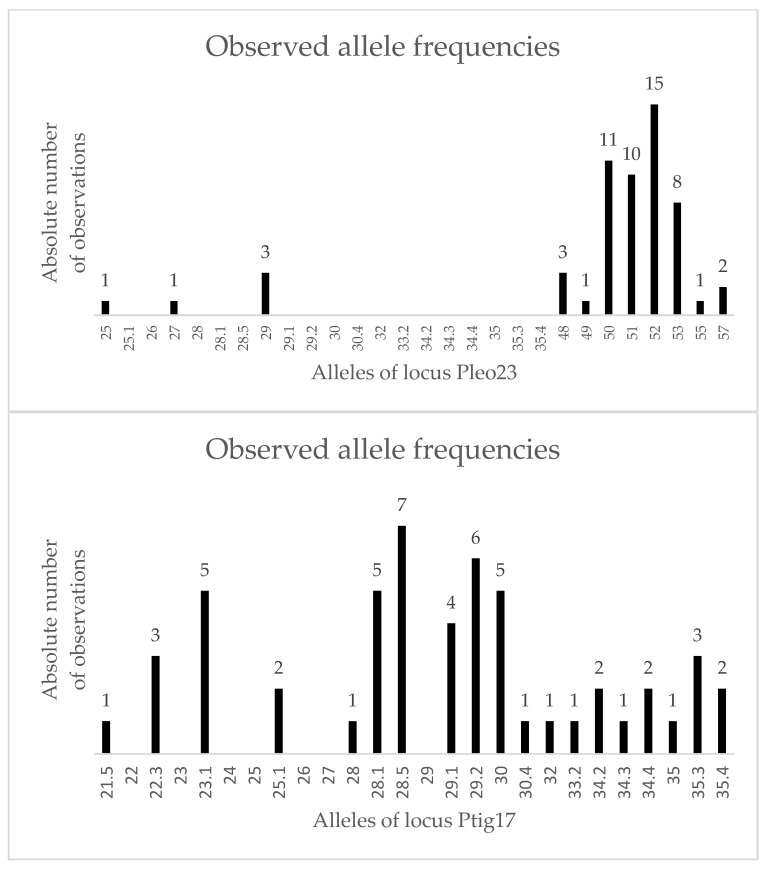
Allele frequencies (absolute number on Y-axis) for alleles observed for *P. pardus* individuals in the most polymorphic loci Ptig17 and Pleo23 (*n* = 30).

In total, 30 individuals were analyzed using the STR multiplex assays [56,57], resulting in 30 unique STR profiles. Each profile consisted of 18 variable polymorphic loci (Figure 2 and Figure 3) and a sex determination system. The statistical evaluation was done using software STRAF, v2.2.2 [58]. Allele frequencies and full forensic parameters are provided in Supplementary Materials S1 and S2 (SM_S1, SM_S2).

**Figure 4:**
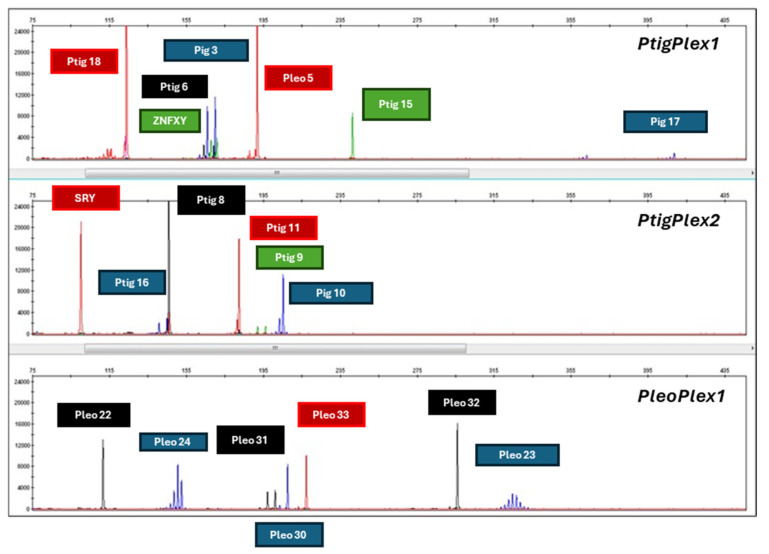
An example of the resulting DNA profiles for a male individual of *P. pardus*, including labels indicating which electropherogram peaks correspond to each locus.

The optimal DNA input concentration for STR-based individual analysis was determined to be ~10 pg of nDNA (example DNA profile of male *P. pardus* is shown in Figure 4, and the corresponding alleles are shown in Table 2). Four STR loci (Ptig15, Ptig18, Ptig8, and Ptig11) were found to be monomorphic, yielding alleles 11.1, 3, 6.1, and 15, respectively, in all tested individuals. Additionally, *P. p. kotiya* subspecies yielded monomorphic status in STR loci Ptig6 (allele 8) and Pleo2 (allele 13). Furthermore, Table 3 presents the repeat structure and flanking-region differences between allele 7 of *P. tigris* and allele 6.1 of *P. pardus*. Notably, the *P. pardus* allele contains a single-base insertion in the 3′ flanking region, which was consistently detected in all sequenced individuals (*n* = 12).

## 4. Discussion

Reliable species and individual identification are fundamental to species protection. In case of the leopard, the urgency is heightened due to the declining trend of the overall population, causing patchier distribution, their involvement in human–wildlife conflict, and the continuing demand for body parts in the illegal wildlife trade. Leopards are among the most exploited felids globally, with thousands of skins, bones, and derivatives estimated to enter the illegal market annually, as the species is of growing forensic concern due to the substitution of its bones for tiger in TCM [11,13,14,19,20,21,22,23].

Traditional morphological approaches are often ineffective for identifying processed wildlife products, where molecular tools provide a valuable and reliable complementary solution for forensic species identification [21,38,39]. For instance, combining morphological analyses of hair with molecular identification can improve the ability of forensic experts to provide evidence provide species-level evidence in support of justice and more effectively combat wildlife crime [28]. In the case of leopards, molecular identification has previously been attempted using species-specific primers [48,49,59,60]. However, some of these assays have proven unreliable [49], and the reliance on species-specific amplicons larger than 220 bp [48,49] is problematic, particularly when working with forensic or wildlife monitoring samples that are often low-yielding or highly degraded. Another limitation of published species-specific primers is the testing of specificity beyond sympatrically occurring species [60].

The *Ppar Qplex* developed in this study addresses key challenges in the forensic analysis of seized wildlife products, which frequently yield low amounts of DNA and are tested across 18 Feliformia species and 2 outgroup species. The *Ppar Qplex* assay targets fragments of Cyt b (164 bp), enabling species-level identification of *P. pardus* with high specificity even from limited template material, while the Feliformia-targeted nuclear marker provided accurate DNA quantification, essential for reliable downstream genotyping. Including an artificial internal positive control further enhanced assay performance by detecting PCR inhibition, a common issue in degraded or processed samples such as hides, hair, or powdered bone. Moreover, the presented STR multiplex demonstrates high sensitivity and robustness across various conditions, and the assay provides a strong foundation for forensic casework. While there is a multiplex qPCR assay available that differentiates between several species of Feliformia, including *P. tigris*, *P. leo*, *P. pardus*, and *A. jubatus* based on melting temperatures [45], this system is unable to detect low quantity/quality template DNA and is unable to quantify the presented sample. Comparable amplification-based qPCR assays have been successfully applied to lion and tiger bones [44] and lynx feces [46]. Our adaptation of amplification-based quantitative real-time PCR extends this approach to leopard, enabling species confirmation and DNA quantification. Beyond law enforcement, a rapid, simple, species-specific, and individual identification tool can help with leopard monitoring and detection from low-yielding, degraded, and mixed samples. Expanding toward source materials such as scent marks, scats, or urine detected in the wild is beneficial for species conservation and monitoring, as proven in species such as *P. tigris* [61]. However, the dataset of tested degraded samples remains limited; although fecal samples were included, which are typically considered degraded DNA sources due to fragmentation, inhibitors, and environmental exposure, future validation on a wider range of highly degraded wildlife products would further strengthen the assay’s forensic applicability.

In addition to the qPCR system, we demonstrate the applicability of the *Pleo STRplex* [57] and *Ptig STRplex* [56] kits as robust genetic tools for conservation and forensic applications within the genus *Panthera*, which includes lions, tigers, jaguars, and leopards. This broad utility is due to the kits targeting specific short tandem repeats (STRs) that are conserved across these species, although some loci exhibit limited variability due to monomorphic status. The degree of monomorphism can vary across *Panthera* species and subspecies [57]. For example, *P. p. kotiya* has shown monomorphic status at two additional STR loci, which remain polymorphic in other *P. pardus* subspecies. This likely reflects the effects of long-term geographic isolation and reduced genetic diversity associated with the endemic status of the Sri Lankan leopard [62].

Importantly, universal STR marker kits provide a substantial advantage over species-specific DNA assays. Their cross-species utility enables reliable analysis even when the exact species origin of a sample is unknown, streamlining laboratory workflows and broadening their applicability [46,56,57,63,64]. This is particularly valuable in forensic science (e.g., identifying individuals from confiscated wildlife products such as tiger bones or leopard skins without prior species knowledge) [65], conservation genetics as shown on other *Panthera* species [66] (e.g., assessing population structure and genetic diversity to guide conservation strategies), and zoological research (e.g., confirming species identity or investigating parentage in captive breeding programs [67]). By enabling efficient and cost-effective testing across a broader spectrum of samples, these universal STR multiplexes transform the analytical process and establish themselves as powerful tools for genetic research and conservation within the genus *Panthera*.

## 5. Conclusions

The presented multiplex qPCR assay and STR system provide a robust framework for addressing the intertwined challenges of species protection, wildlife conflict, and wildlife trade. By enabling both species and individual identification from a wide range of sample types, including low-yielding samples, these tools strengthen law enforcement capacity, support conservation monitoring, and ultimately contribute to the survival of *P. pardus*.

## Figures and Tables

**Table 1:** Primers and probes used in *Ppar Qplex* (* newly designed primers).

Primer/Probe Name	Final Concentration	Sequence (5′-3′)	PCR Product Size	Specificity	TaqMan Probe Fluorescent Label	Design
	(mM)		(bp = Base Pairs)			
qPparM_F	5	AGACATGGAACATTGGAGTC	164 bp	*Cyt b* (mtDNA)	---	*
qPparM_R	5	TCAGATTCATTCTACTAGGTCAATC			---	*
qPparM_probe	1.7	CAACCGTAATTACCAACCTCC		probe	VIC	*
qPparC_F	5	CTGCTAGGTTTAGCGCGTGAC	261 bp	IPC	---	[46]
qPparC_R	5	GGGGACCATGCTTGCG			---	[46]
qPparC_probe	1.7	TGCACGATTCAAGCACGAT		probe	NED	[46]
qPparN_F	3.3	AGTCCACTTCTCATTGCCCCTT	132 bp	PLP (nDNA)	---	[46]
qPparN_R	3.3	ACCTTCCCTGAGTTCTCCATACC			---	[46]
qPparN_probe	1.7	CTCACCAGACCTGTTAGGA		probe	6-FAM	[46]

**Table 2:** Resulting allele calls for a male individual of *P. pardus*.

*P. pardus*		
Multiplex	Locus	Alleles
PtigPlex1	Ptig3	12, 13
	Ptig17	28.1, 35.4
	Ptig15	11.1, 11.1
	Ptig6	7, 8
	Ptig18	3, 3
	Ptig5	10, 10
	ZNFXY	M
PtigPlex2	Ptig16	13, 14
	Ptig10	23, 23
	Ptig9	14, 15
	Ptig8	6.1, 6.1
	Ptig11	15, 15
	SRY	M
PleoPlex1	Pleo24	19, 20
	Pleo30	22, 22
	Pleo23	56, 57
	Pleo22	7.3, 7.3
	Pleo31	21, 22
	Pleo32	14, 14
	Pleo33	15, 15

**Table 3:** STR sequence difference of *P. tigris* and *P. pardus* for the selected locus Ptig 8. Please note the single base insertion in the flanking region of *P. pardus* allele 6.1. The same insertion was found across all individuals sequenced (*n* = 12).

	*P. tigris*	*P. pardus*
***Ptig 8*** **(ATCTAT)n (ATC)n**	alelle 7 — 6 × (ATCTAT) + 2 × (ATC)	alelle 6.1 **5** × (ATCTAT) + 2 × (ATC) + 1 bp insertion
	GCTGAT **ATCTAT ATCTAT ATCTAT ATCTAT ATC ATCTAT ATC ATCTAT** ATTTTT CCCCC TCTC	GCTGAT **ATCTAT ATCTAT ATCTAT ATC ATCTAT ATC ATCTAT** ATTTTT ***T***CCCC CTC

## Data Availability

The data presented in this study are available upon request from the corresponding author due to the funding agency’s request.

## Supplementary Materials

The following supporting information can be downloaded at: https://www.mdpi.com/article/10.3390/genes17010045/s1, SM_S1: forensic parameters; SM_S2: allele frequencies.
